# Long non-coding RNA *UCA1* induces non-T790M acquired resistance to EGFR-TKIs by activating the AKT/mTOR pathway in *EGFR*-mutant non-small cell lung cancer

**DOI:** 10.18632/oncotarget.4361

**Published:** 2015-06-08

**Authors:** Ningning Cheng, Weijing Cai, Shengxiang Ren, Xuefei Li, Qi Wang, Hui Pan, Mingchuan Zhao, Jiayu Li, Yishi Zhang, Chao Zhao, Xiaoxia Chen, Ke Fei, Caicun Zhou, Fred R. Hirsch

**Affiliations:** ^1^ Department of Medical Oncology, Shanghai Pulmonary Hospital, Tongji University, Tongji University Medical School Cancer Institute, Shanghai, P. R. China; ^2^ Department of Lung Cancer and Immunology, Shanghai Pulmonary Hospital, Tongji University, Tongji University Medical School Cancer Institute, Shanghai, P. R. China; ^3^ Department of Thoracic Surgery, Shanghai Pulmonary Hospital, Tongji University, Tongji University Medical School Cancer Institute, Shanghai, P. R. China; ^4^ Department of Medical and Pathology, University of Colorado Cancer Center, Aurora, Colorado, USA

**Keywords:** UCA1, EGFR-TKIs, acquired resistance, non-small cell lung cancer

## Abstract

The aim of this study was to explore the role of long non-coding RNA *UCA1* (urothelial cancer-associated 1) in acquired resistance to epidermal growth factor receptor tyrosine kinase inhibitors (*EGFR*-TKIs) in *EGFR*-mutant non-small cell lung cancer (NSCLC). In our study, *UCA1* expression was significantly increased in lung cancer cells and patients with acquired resistance to *EGFR*-TKIs. Over-expression of *UCA1* was significantly associated with a shorter progression-free survival (PFS) [13.0 *vs*. 8.5 months, *P* < 0.01] in tumors with respond to *EGFR*-TKIs. The significant relationship was not observed in patients with T790M mutation (10.5 *vs*. 12.0 months, *P* = 0.778), but in patients with non-T790M (19.0 vs. 9.0 months, *P* = 0.023). *UCA1* knockdown restored gefitinib sensitivity in acquired resistant cells with non-T790M and inhibited the activation of the AKT/mTOR pathway and epithelial-mesenchymal transition (EMT). The mTOR inhibitor was effective in *UCA1*-expressing cell PC9/R. Inhibiting mTOR could change the expression of *UCA1*, although there was no significant difference. In conclusion, the influence of over-expression of *UCA1* on PFS for patients with acquired resistance to *EGFR*-TKIs was from the subgroup with non-T790M mutation. *UCA1* may induce non-T790M acquired resistance to *EGFR*-TKIs by activating the AKT/mTOR pathway and EMT.

## INTRODUCTION

Epidermal growth factor receptor (*EGFR*) tyrosine kinase inhibitors (*EGFR*-TKIs) such as gefitinib and erlotinib are first-line treatments for advanced non-small cell lung cancer (NSCLC) harboring *EGFR*-activating mutations, and have been reported to improve the clinical outcome and quality-of-life of patients with this malignancy [1-4]. However, acquired resistance invariably develops, and the majority of *EGFR*-mutated NSCLCs that respond to *EGFR*-TKIs develop acquired resistance after approximately 12 months [5, 6]. The most common mechanism whereby acquired resistance to EGFR-TKIs develops is a secondary *T790M* mutation [7]. As well as this mechanism, *c-MET* amplification(5-10%), *PIK3CA* mutations(∼5%), *BRAF* mutations(∼1%) and small-cell lung cancer transformation(∼5%) are also associated with acquired resistance to TKIs [8-10]. However, the mechanisms responsible for about 30% of cases of acquired resistance to *EGFR*-TKIs are still unknown [10].

Long non-coding RNAs (lncRNAs) are a group of non-coding transcripts of more than 200 nt that are involved in cell apoptosis, tumor invasion, metastasis, and drug resistance [11, 12]. Multiple studies have indicated that lncRNAs, including *H19* [13], *CUDR* [14], and *AK126698* [15] are related to chemotherapy resistance. In a previous study [16], we compared the expression of lncRNAs in gefitinib-sensitive and gefitinib-resistant human lung cancer cells by lncRNA microarray analysis, and found that some lncRNAs, including *UCA1* (urothelial cancer-associated 1), were up-regulated in resistant cells.

In an effort to overcome resistance, we have investigated the molecular mechanisms of acquired resistance in epigenetic genetics. In the present study, we sought to determine whether the lncRNA *UCA1* can induce acquired resistance to *EGFR*-TKIs via cell apoptosis and activation of the PI3K/AKT/mTOR pathway in *EGFR*-mutant lung cancer.

## RESULTS

### Over-expression of *UCA1* was correlated with acquired resistance to *EGFR*-TKIs

To identify the mechanisms of acquired resistance to *EGFR*-TKIs, we conducted microarray expression profiling of lncRNAs/mRNA for PC9 and PC9/R cells. *UCA1* was found to have a high expression level in PC9/R cells with acquired resistance to gefitinib [16]. To validate the analysis of lncRNAs profiles, we assessed the mRNA expression of *UCA*1 by RT-PCR in lung cancer cell lines and patients with *EGFR*-mutant NSCLC. A total of 89 patients were enrolled in this study. Of them, the clinical characteristics of the 84 patients with *EGFR*-mutant NSCLC who had enough samples obtained from either before *EGFR*-TKIs treatment or after development of acquired resistance to *EGFR*-TKIs were shown in Table 1 and that of the other 5 patients who had matched samples were shown in Table 2.

**Table 1:** Clinical characteristics of the 47 patients with *EGFR*-mutant NSCLC(BT group) and 37 with acquired resistance to *EGFR*-TKIs(AR group)

Clinical characteristics	BT group N = 47 (%)	AR group N = 37 (%)
Age:		
<65 years	33 (70.2%)	27 (73.0%)
≥65 years	14 (29.8%)	10 (27.0%)
Sex:		
Male	18 (38.3%)	21 (56.8%)
Female	29 (61.7%)	16 (43.2%)
EGFR:		
19DEL	27 (57.4%)	9 (24.3%)
L858R	20 (42.6%)	8 (21.6%)
T790M	-	20 (54.1%)
Stage:		
IIIB	2 (4.3%)	8 (21.6%)
IV	45 (95.7%)	29 (78.4%)
Smoking:		
Never	39 (83.0%)	28 (75.7%)
Ever	8 (17.0%)	9 (24.3%)
Histology:		
Adenocarcinoma	44 (93.6%)	35 (92.9%)
Non-adenocarcinoma	3 (6.4%)	2 (5.4%)
UCA1:		
Low	34 (72.3%)	22 (59.5%)
High	13 (27.7%)	15 (40.5%)
*EGFR*-TKIs:		
Gefitinib	39 (83.0%)	17 (45.9%)
Erlotinib	8 (17.0%)	20 (54.1%)

EGFR: epidermal growth factor receptor; TKI: tyrosine kinase inhibitor; UCA1: urothelial carcinoma-associated 1; BT: before treatment; AR: acquired resistance.

**Table 2:** UCA1 is up-regulated in human EGFR-mutant NSCLC specimens from individuals with acquired resistance to *EGFR*-TKIs

ID	Age	Sex	Tumor type	EGFR mutation		TKI	Response	PFS (months)	2^−ΔCt,^ UCA1	
				BT	AR				BT	AR
1	49	M	AC	L858R	L858R	Erlotinib	PR	16.0	0.008	0.042
2	67	F	AC	19DEL	19DEL	Gefitinib	PR	17.3	0.105	0.204
3	63	F	AC	19DEL	19DEL/T790M	Erlotinib	SD	13.9	0.040	0.259
4	54	F	AC	19DEL	19DEL	Gefitinib	PR	13.0	0.031	0.357
5	37	M	NSCLC	L858R	L858R/T790M	Erlotinib	SD	8.5	0.004	0.188

Clinical characteristics in the 5 paired EGFR-mutant NSCLC specimens were obtained from patients both before treatment and upon acquired resistance to treatment with erlotinib or gefitinib.

AC: adenocarcinoma; AR: acquired resistance; BT: before treatment; EGFR: epidermal growth factor receptor; F: female; M: male; NSCLC: non-small cell lung cancer; PFS: progression-free survival; PR: Partial response; SD: stable disease; TKI: tyrosine kinase inhibitor; UCA1: urothelial carcinoma-associated 1.

**Figure 1:**
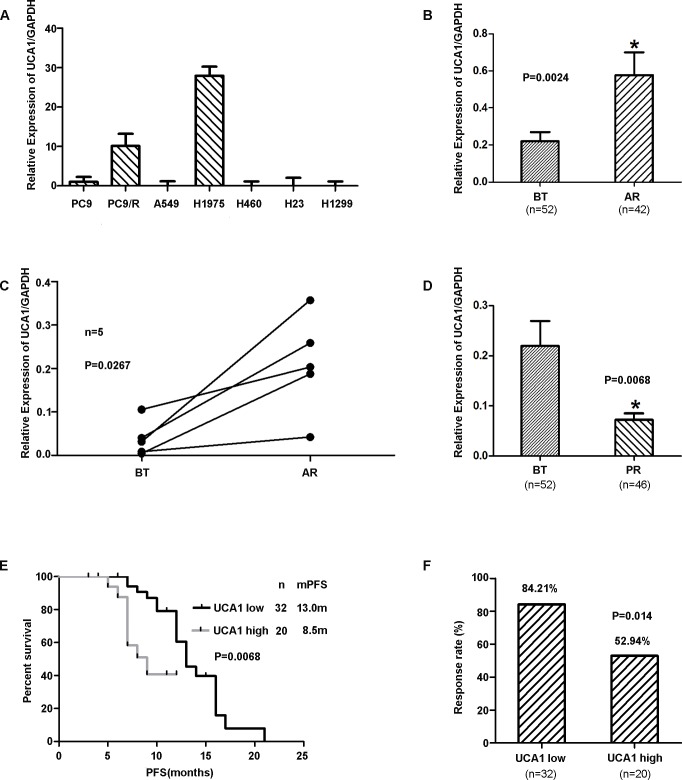
**A.**The expression of UCA1 in lung cancer cells. Over-expression of UCA1 was observed in lung cancer cells with acquired resistance (PC9/R and H1975cells); however, in primary resistant cells (A549, H460, H23 and H1299), UCA1 was down-regulated (*P* < 0.01). **B.** UCA1 expression levels in lung cancer tissues assessed by qRT-PCR in patients with *EGFR*-TKI-sensitive NSCLC (before treatment) and patients who developed acquired resistance to *EGFR*-TKIs. **C.** UCA1 expression levels assessed in 5 paired *EGFR*-mutant patients both before treatment and upon acquired resistance to *EGFR*-TKIs. **D.** UCA1 expression levels were assessed in *EGFR*-TKIs before treatment and primary resistance. **E.** Progression-free survival (PFS) in patients with high and low UCA1 expression levels before *EGFR*-TKI treatment. **F.** The objective response rate (ORR) in patients with high and low UCA1 expression levels before *EGFR*-TKI treatment. BT: before treatment; AR: acquired resistance; PR: primary resistance; PFS: progression-free survival.

Over-expression of *UCA1* was observed in lung cancer cells with acquired resistance (PC9/R and H1975) [*P* < 0.01] (Figure 1A). And *UCA1* mRNA expression level in patients who developed acquired resistance to *EGFR*-TKIs was significantly higher than in the baseline group with *EGFR*-TKI-sensitive NSCLC (0.58 ± 0.05 *vs* 0.21 ± 0.05, *P* = 0.0024; Figure 1B). We also measured the mRNA expression of *UCA1* by RT-PCR in 5 matched *EGFR*-mutant NSCLC specimens, including 2 with T790M and 3 without T790M(Table 2) (Figure 1C), both before treatment (BT) with *EGFR*-TKIs and after the development of resistance to TKIs, and found that *UCA1* expression was up-regulated in patients with acquired resistance. Whereas it was down-regulated in patients with primary resistance(0.072 ± 0.013 *vs* 0.21 ± 0.05, *P* = 0.0068; Figure 1D). On the basis of the *UCA1* expression before treatment with *EGFR*-TKIs, the patients were divided into a high expression group (n = 20) and a low expression group (n = 32), depending on whether they were above or below the cut-off value 2^−ΔCt^ = 0.068 (Supplementary Table S1). When progression free survival(PFS) was assessed, patients in the high *UCA1* expression group had a significantly poorer prognosis than those in the low expression group (median PFS 8.5m *vs* 13.0m, *P* = 0.0068; Figure 1E). The objective response rate (ORR) in the high *UCA1* expression group was significantly lower than in the low expression group (52.94% *vs* 84.21%, *P* = 0.014; Figure 1F). Univariate analysis of PFS revealed that the expression level of *UCA1* and age were prognostic indicators (Table 3), while multivariate analysis indicated that the *UCA1* expression level and age were independent prognostic factors for PFS in patients with *EGFR*-TKI-sensitive NSCLC. Therefore, we hypothesized that *UCA1* may play an important role in acquired resistance to *EGFR*-TKIs and influence the efficacy of *EGFR*-TKIs.

**Table 3:** Univariate and multivariate analysis for progression-free survival (PFS)

Factors	Univariate analysis		Multivariate analysis	
	HR (95% CI)	*P*	HR (95% CI)	*P*
Age (<65/≥65 years)	0.298 (0.089-0.999)	0.05	0.296 (0.088-0.993)	0.049
Sex (male/female)	0.591 (0.265-1.318)	0.199		
Smoking (never/ever)	1.944 (0.559-6.766)	0.296		
EGFR (19DEL/L858R)	1.167 (0.525-2.593)	0.705		
Histology (adenocarcinoma/non-adenocarcinoma)	1.15 (0.268-4.94)	0.851		
Stage (IIIB/IV)	2.702 (0.612-11.927)	0.189		
UCA1 (low/high)	3.339 (1.281-8.699)	0.015	0.308 (0.111-0.851)	0.023
*EGFR*-TKIs (gefitinib/erlotinib)	0.946 (0.381-2.344)	0.904		

CI: confidence interval; EGFR: epidermal growth factor receptor; HR: hazard ratio; TKI: tyrosine kinase inhibitor; UCA1: urothelial carcinoma-associated 1.

### The impact of over-expression of *UCA1* on PFS for patients with acquired resistance to EGFR-TKIs was from T790M-negative subgroup

**Figure 2:**
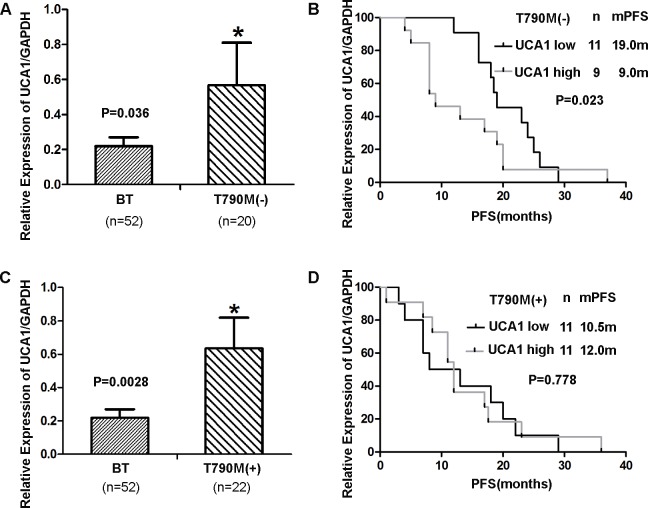
**A., C.** UCA1 expression levels assessed in patients treated with *EGFR*-TKI-sensitive NSCLC (baseline group) and patients who were without T790M and with T790M mutations. **B.**, **D.** PFS in patients with acquired resistant patients who were without T790M mutations and with T790M mutations.

We observed the expression level of *UCA1* was significantly higher in patients with acquired resistance to *EGFR*-TKIs regardless of the status of T790M mutation than in BT group (subgroup without T790M, 0.57 ± 0.24 *vs* 0.21 ± 0.05, *P* = 0.036; Figure 2A) (subgroup with T790M, 0.64 ± 0.18 *vs* 0.21 ± 0.05, *P* = 0.0028; Figure 2C). However, the expression of *UCA1* was significantly associated with PFS in only patients without T790M mutations (P = 0.023; Figure 2B). The relationship was not observed in patients with T790M mutations (*P* = 0.778; Figure 2D). Therefore, we hypothesized that *UCA1* may play an important role in acquired resistance to *EGFR*-TKIs in patients without T790M mutations.

### *UCA1* inhibition restored gefitinib sensitivity in acquired resistant cell lines without T790M *in vitro* and *in vivo*

**Figure 3:**
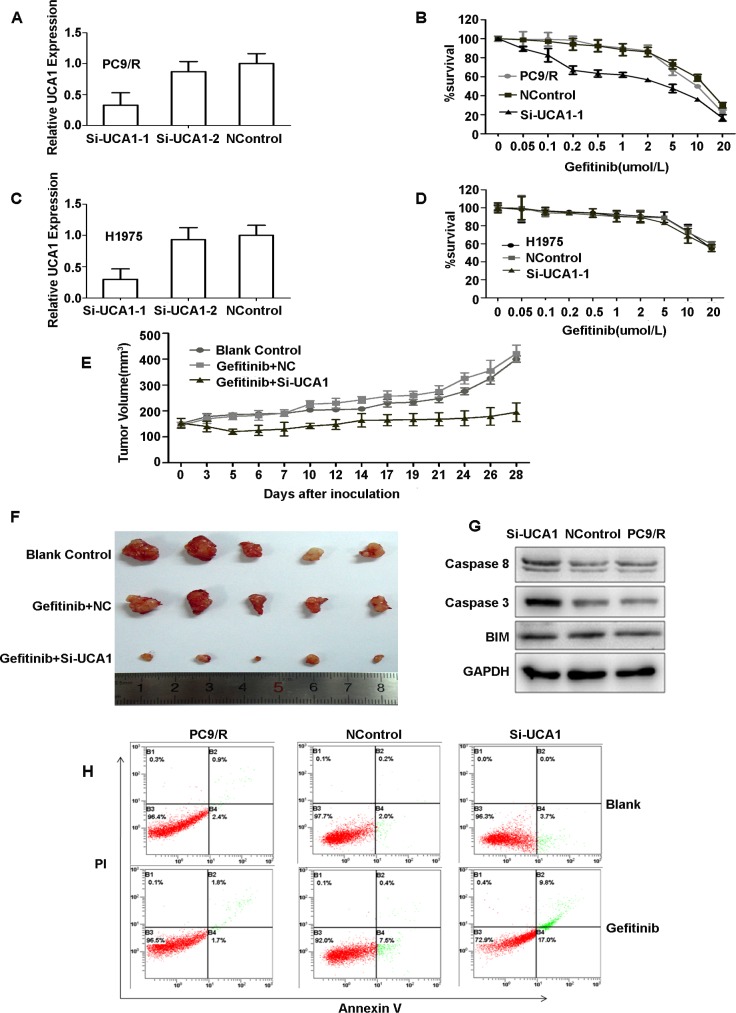
**A., C.** qRT-PCR detection of UCA1 expression in PC9/R and H1975 cells after silencing of UCA1 by si-RNA. The relative expression of UCA1 was 65% lower with si-UCA1 than with the negative control. **B.**, **D.** The sensitivity to gefitinib of PC9/R and H1975 cells was detected by CCK-8 (Cell Counting Kit-8). Cells were exposed to various concentration of gefitinib for 72hours. Inhibiting the UCA1 gene resulted in an approximately 2-fold decrease in the gefitinib IC_50_ in PC9/R cells (IC_50_insi-UCA1-PC9/R and PC9/R cells, 7μmol/L and 15μmol/L, respectively), but the IC_50_ in H1975 cells was not changed (IC_50,_ 20μmol/L). **E.**, **F.** Tumor volumes of PC9/R cells transfected with si-UCA1, negative control (NC) and blank control after gefitinib treatment *in vivo*. After 4 weeks, tumor weights were represented as means ± SD. Western blot analysis **G.**, caspase 3, caspase 8, and BIM(Bcl-2 interacting mediator of cell death). **H.** Gefitinib-induced apoptosis in PC9/R cells was demonstrated by flow cytometric analysis. Cells were treated with gefitinib for 72hours and then analyzed for early apoptotic cells (bottom right quadrant) and late apoptotic cells (top right quadrant). The percentages of cells in the two quadrants are shown.

To assess the role of *UCA1* in acquired resistance to *EGFR*-TKIs, the effect of *UCA1* on cell proliferation and apoptosis was investigated. The silencing capacity of si-UCA1 was evaluated by using qRT-PCR. Si-UCA1-1 showed an optimal effect in comparison with si-UCA1-2 and the negative control (NC) (Figures 3A, 3C). After inhibiting the *UCA1* gene, the sensitivity to gefitinib was partly restored in PC9/R cells, but this effect was not observed in H1975 cells (Figures 3B, 3D).

To further validate the effect of *UCA1* on *EGFR*-TKI-resistant NSCLC cells *in vivo*, we established a gefitinib-resistant PC9/R model. Consistent with previous observations, we found that gefitinib plus si-UCA1 treatment inhibited tumor growth, but these changes were not observed in blank control and gefitinib plus negative control (NC)-treated tumors (Figure 3E, 3F). These results were consistent with our clinical data and further confirmed our hypothesis.

As refractoriness to apoptosis induced by *EGFR*-TKIs is one of the major features of resistance to targeted therapy in NSCLC, the effect of *UCA1* on cell apoptosis was examined. We observed that *caspase 3* and *caspase 8* (the activation of which may be involved in cell apoptosis) were both increased by transfecting si-UCA1 (Figure 3G). A significantly higher percentage of apoptotic cells were found in si-UCA1-treated cells (26.8%) in comparison with those transfected with the negative control (7.9%) (Figure 3H). Taken together, these results indicate that inhibition of UCA1 induces apoptosis in cells resistant to *EGFR*-TKIs.

### *UCA1* may promote activation of the PI3K/AKT/mTOR pathway and EMT

**Figure 4:**
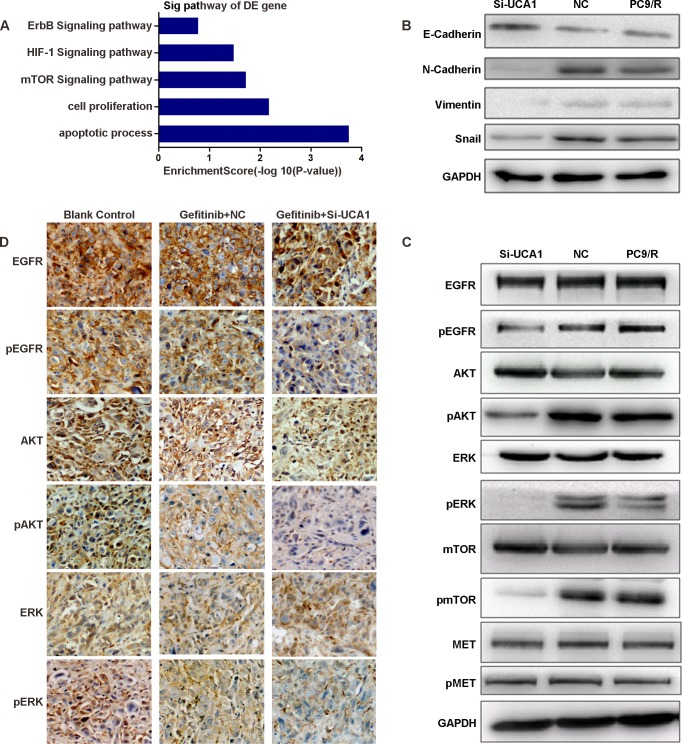
**A.** Signaling pathways of differentially expressed RNAs. **B.**, **C.** Western blot analysis the proteins of epithelial-mesenchymal transition(EMT) and EGFR and its downstream proteins in si-UCA1-treated and negative control (NC)-treated PC9/R cells and non-treated PC9/R cells. **D.** Immunohistochemistry (IHC) revealed that pEGFR, pAKT and pERK were detected in control and gefitinib plus NC-treated tumors, but not in gefitinib plus si-UCA1-treated tumors. The experiments were repeated at least 3 times, and a representative one is shown.

Based on the latest KEGG (Kyoto Encyclopedia of Genes and Genomes) database, a pathway analysis was performed for differentially expressed mRNAs in both PC9 and PC9/R cell lines before *UCA1* knockdown. Among these, enriched pathways relating to mTOR signaling suggested a role in acquired resistance to *EGFR*-TKIs (Figure 4A). PI3K/AKT/mTOR and ERK are two crucial downstream signaling pathways for EGFR [17]. Therefore, to explore the underlying molecular mechanisms of *EGFR*-TKI resistance, we assessed whether *UCA1* affects the expression of crucial proteins in these signaling pathways. Western blot analysis showed that the expressions of phospho-EGFR (pEGFR), phospho-AKT (pAKT), phospho-ERK (pERK), and phospho-mTOR (pmTOR) were positively correlated with the expressions of *UCA1* among si-UCA1-treated PC9/R cells and negative control (NC)- treated PC9/R cells and non- treated PC9/R cells (Figure 4C), but the expressions of total EGFR, AKT, ERK, mTOR, MET, and pMET were not changed.

The mTOR inhibitor was effective in *UCA1*-expressing cell PC9/R, with IC_50_ of 8.3μmol/L. Inhibiting mTOR could change the expression of *UCA1*, although there was no significant difference. Therefore, we considered that *UCA1* may have an impact on mTOR pathway(Supplementary Figure S4).

To confirm that si-UCA1 can inhibit PI3K/AKT and ERK signaling pathways *in vivo*, we assessed the expression of *EGFR*, pEGFR, AKT, pAKT, ERK, and pERK. Immunohistochemistry (IHC) revealed that the expression levels of pEGFR, pAKT and pERK were significantly higher in blank control and gefitinib plus NC-treated tumors than in gefitinib plus si-UCA1-treated tumors (Figure 4D) [Supplementary Figure S1]. Thus, these *in vivo* data complemented the functional studies of *UCA1 in vitro* in demonstrating that *UCA1* is capable of promoting EGFR-TKI resistance *in vivo*. Accordingly, si-UCA1 may overcome gefitinib resistance which is not caused by T790M.

Epithelial-mesenchymal transition (EMT) plays a critical role in resistance to *EGFR*-TKIs, with a decrease of epithelial markers such as E-cadherin and an increase of mesenchymal markers such as vimentin [18, 19]. An association between *UCA1* and markers of EMT was observed. The results indicated that knockdown of *UCA1* enhanced the expression of E-cadherin, whereas the expression of vimentin, Snail, and N-cadherin were attenuated (Figure 4B).

Taken together, these findings indicate that the expression of *UCA1* is positively correlated with pEGFR, pAKT, pERK and pmTOR, but is not related to MET. Thus, *UCA1* may activate AKT/mTOR, ERK pathways and EMT to promote resistance to gefitinib.

## DISCUSSION

The majority of patients with *EGFR*-mutant lung cancers eventually develop acquired resistance to *EGFR*-TKIs [5, 6]. However, the mechanisms responsible for most patients with non-T790M acquired resistance to *EGFR*-TKIs are still unknown [10]. It is known that epigenetics especial non-coding RNAs play a key role in *EGFR*-TKIs resistance [20, 21]. In previous studies, we reported that miR-21, miR-214 and miR-200 are involved in both acquired resistance and primary resistance to *EGFR*-TKIs [22, 23]. However, lncRNAs can connect to transcription sites and regulate both the expression of alleles and a long fragment, whereas coding genes and micro-RNAs have no such functions, which suggests that lncRNAs may be better epigenetic regulators in controlling performance [24]. Moreover, the function of lncRNAs in the development of acquired resistance to *EGFR*-TKIs is still unknown. Therefore, we explored the role and possible molecular mechanism of lncRNAs in acquired resistance to *EGFR*-TKIs in NSCLC.

To gain insight into the molecular mechanisms of *EGFR*-TKI resistance, we compared the expression profile of lncRNAs between gefitinib-sensitive and gefitinib-resistant human lung cancer cells by lncRNA microarray and found that thousands of lncRNAs were up-regulated in gefitinib-resistant cells [16]. In addition, through bioinformatic analysis, we identified that the lncRNA-UCA1 is related to cell apoptosis, proliferation, and chemoresistance. *UCA1* was first identified in bladder cancer cells and is involved in bladder cancer invasion and progression [25]. As it was observed that *UCA1* is up-regulated in liver, colon and stomach cancers, it may be a biomarker for the diagnosis of these cancers [26, 27]. Of note, *UCA1* has been shown to be up-regulated in lung cancer and induce chemoresistance [27].

One of important findings of this study was that over-expression of *UCA1* in lung cancer cells and patients with acquired resistance to *EGFR*-TKIs. Our clinical data demonstrated that *UCA1* expression levels were significantly higher in *EGFR*-mutant NSCLC patients who developed acquired resistance to *EGFR*-TKIs compared with before treatment levels, suggesting that high expression of *UCA1* may be a mechanism of resistance to *EGFR*-TKIs. Meanwhile, the high expression of *UCA1* was correlated with the poorer prognosis than those in the low expression group. Univariate and multivariate analysis of PFS revealed that *UCA1* and age were independent prognostic factors. However, we found that over-expression *UCA1* was not significantly associated with PFS for patients with T790M acquired resistance to *EGFR*-TKIs, although the significant high expression level of *UCA1* in NSCLC with acquired resistance regardless of T790M status was observed. We therefore hypothesized that high expression of *UCA1* may be one of the mechanisms of acquired resistance to *EGFR*-TKIs in *EGFR*-mutant NSCLC without T790M. We also validated the effect of *UCA1* on *EGFR*-TKI resistance in NSCLC cells *in vitro* and *in vivo*.

*In vitro*, we observed that *UCA1* knockdown can partly restore the sensitivity of PC9/R cells (19DEL, without T790M and MET amplification), but this change was not observed in H1975 cells (L858R/T790M). *In vivo*, we also found that gefitinib in combination with si-UCA1 inhibited tumor growth in gefitinib-resistant PC9/R model. The data were consistent with the cell experiments and further confirmed our hypothesis.

Our study also showed that *UCA1*-mediated acquired resistance to gefitinib may occur through activation of the AKT/mTOR pathway and EMT. Previous studies have implicated activation of the PI3K/AKT/mTOR and ERK pathways as well as EMT in resistance to *EGFR*-TKIs [28-30]. Subsequently, other studies have reported that *UCA1* can promote cell proliferation and invasiveness by activating the PI3K/AKT pathway [31-33]. These studies confirm the validity of our results. In addition, the effects of *UCA1* on cell proliferation and invasiveness in our study were similar to those of *Nodal* in breast cancer. *Nodal* has been demonstrated to promote invasiveness and metastasis in breast cancer cells via EMT and ERK pathway activation [34]. Besides, Li ZK et al. have reported that *UCA1* promotes glycolysis through the mTOR pathway. They considered *UCA1* was associated with the mTOR pathway [35]. Therefore, base on our data, we considered *UCA1* may be associated with AKT/mTOR and ERK pathways and EMT. Further investigations will be required to elucidate the mechanisms by which *UCA1* regulates the AKT/mTOR signaling pathway and EMT. We therefore hypothesized that *UCA1*-mediated acquired resistance in the absence of T790M mutations is likely to be related to activation of the AKT/mTOR and ERK pathways.

Recently, new generation *EGFR*-TKIs such as CO-1686 and AZD9291 have been found to be irreversible inhibitors that can overcome acquired resistance caused by T790M [36-39]. To date, however, patients without T790M mutations who develop acquired resistance to *EGFR*-TKIs have no effective treatment, as the mechanisms of acquired resistance remain unclear. Some studies have reported that the histone lysine-specific demethylase 1(LSD1) enzyme EZH2 may be a new “druggable” epigenetic target [40-42]. Therefore, we consider that *UCA1* may play a key role in overcoming non-T790M acquired resistance to *EGFR*-TKIs by functioning as a new epigenetic regulator in NSCLC.

In conclusion, we have identified that *UCA1* over-expression was significantly associated with poor outcome of NSCLC patients with acquired resistance to *EGFR*-TKIs and the impact of over-expression of *UCA1* on PFS for patients with acquired resistance to *EGFR*-TKIs was from non-T790M subgroup. We consider over-expression of *UCA1* as a novel mechanism by which acquired resistance to *EGFR*-TKIs can develop in *EGFR*-mutant NSCLC patients without T790M mutations. *UCA1* may regulate resistance to gefitinib through activation of the AKT/mTOR pathway and EMT. Further studies will be required to elucidate the precise mechanisms of *UCA1*-mediated acquired resistance.

## MATERIALS AND METHODS

### Cell culture and tissues collection

The human lung adenocarcinoma cell lines PC9 (EGFR exon 19 deletion), H1975 (L858R/T790M), A549 (EGFR wild-type), H460, H23, and H1299 were obtained from American Type Culture Collection (ATCC, Manassas, VA, USA). The gefitinib-resistant cell line PC9/R, which has no T790M and MET amplifications [43], was provided by Shanghai Pulmonary Hospital. All cells were cultured at 37°C in a humidified incubator with 5% CO_2_ in Dulbecco's modified Eagle's medium (DMEM) [Hyclone, Logan, UT, USA] supplemented with 10% fetal bovine serum(FBS) [Sigma Aldrich].

Ninety-four advanced lung adenocarcinoma tissues were collected from NSCLC patients who had either an exon 19 deletion (19DEL) or an exon 21 point mutation (L858R) in their *EGFR*s, and were treated with either gefitinib or erlotinib between January 2012 and December 2013, with the written consent of the patients involved and the approval of the Shanghai Pulmonary Hospital Ethics Committee. All patients had either prolonged stable disease (SD) of more than 6 months or a partial response (PR) to *EGFR*-TKIs therapy and 42 of 89 patients met the established clinical definition of acquired resistance to *EGFR*-TKIs [5]. Five of these 42 patients had available samples obtained before *EGFR*-TKIs treatment and after acquired resistance to *EGFR*-TKIs. Efficacy data were monitored until the end of June 2014. Of these 94 collected samples, 42 were collected from patients after they developed acquired resistance to *EGFR*-TKIs(defined as AR group), other 52 were collected from patients before *EGFR*-TKIs treatment(defined as BT group). Of note, there were 5 matched *EGFR*-mutant NSCLC samples.

We also collected fourty-six primary resistant patients. Primary resistance to *EGFR*-TKI was defined as progression on the first imaging evaluation or SD < 6 months after *EGFR*-TKI treatment in the first setting for patients with NSCLC harboring an activating EGFR mutation.

### Quantitative reverse transcription polymerase chain reaction(qRT-PCR)

Total RNA was extracted from the lung cancer cell lines using TRIzol reagent (TaKaRa, Japan) or from tissue samples using an RNeasy Mini Kit(QIAGEN). The expression of *UCA1* in lung cancer cell lines and tissues was measured by qPCR methodology using SYBR Premix Ex Taq (TaKaRa) and an MX3000P instrument. *UCA1* primers were designed by Sangon Biotech (China). Glyceraldehyde 3-phosphate dehydrogenase(GAPDH) was used as a control. All experiments were performed in triplicate, and the median of each triplicate set of values was used to calculate relative lncRNA concentrations as follows:

ΔCt (Cycle threshold) = Ct_median lncRNA_ − Ct_median GAPDH_

Fold changes were calculated using 2^−ΔΔCt^ methods.

### Si-RNA transfection

PC9/R and H1975 lung cancer cells (2×10^5^) were seeded into each well of 6-well plates and incubated overnight, and then transfected with 100nmol/L of small-interfering (si)-UCA1-1 or si-UCA1-2 and a negative control (NC) purchased from RiboBio (Guangzhou, China) that consisted of Lipofectamine^®^ 2000 transfection reagent (Invitrogen, USA). The target sequence for si-UCA1-1 was as follows:

sense strand, 5′-GCCACCUACAUUAAAGCUAdTdT-3′, antisense strand, 3‘-dTdT CGGUGGAUGUAAUUUCGAU-5′.

Forty-eight hours after transfection, the cells were harvested for real-time PCR or western blot analysis.

### Cell proliferation and apoptosis assays

After transfection, the cells were seeded overnight at a density of 5×10^3^ cells in 96-well plates in DMEM containing 10% FBS, and then exposed to various concentrations of gefitinib for 72hours. 10μLof CCK-8 reagent (Dojingdo Molecular Technology, Japan) was added to the cells for 1hour at 37°C, and the absorbance in each well was measured at 450 nm by an enzyme-labeled instrument.

The PC9/R cells were seeded in 6-well plates for 24hours and then transfected with si-UCA1-1 and the negative control. After gefitinib treatment for 72hours, the cells were trypsinized, washed twice with PBS, and resuspended in binding buffer. They were then stained with Annexin V/PI (Invitrogen, USA) for 15min in the dark at room temperature, and the cell populations were analyzed by a flow cytometer.

### Western blot analysis

Cells were lysed using RIPA protein extraction reagent (Beyotime, Beijing, China) supplemented with phenylmethanesulfonyl fluoride (PMSF) [Riche, CA, USA]. Approximately 25μg of protein extracts were separated by 10% sodium dodecyl sulfate polyacrylamide gel electrophoresis (SDS-PAGE), transferred onto nitrocellulose membranes (Sigma), and incubated with specific antibodies. An enhanced chemiluminescent (ECL) chromogenic substrate was used to visualize the bands. The blots were developed with a chemiluminescence system, and GAPDH was used as a control. All antibodies were purchased from Abcam(Cambridge, UK).

### Xenograft study

For this part of the study, 5-week-old female specific pathogen-free (SPF) nude mice were used. The animal studies were approved by our Institutional Animal Care and Use Committee, and were performed according to institutional guidelines. PC9/R cells were injected into the right flanks of the mice, and gefitinib treatment was started 10 days after the tumor cell inoculation. Gefitinib was administered by oral gavage on 5 days per week at a dosage of 25mg/kg in 1% Tween 80 (Sigma). Si-UCA-1 or the negative control was administered as intratumoral injections.

Tumor sizes were assessed three times per week by a digital caliper. The tumor volumes were determined by measuring their length (l) and width (w) and calculating the volume (V) as follows: V = lw^2^/2. After 30 days, the mice were killed and paraffin-embedded tissues were prepared for immunohistochemical (IHC) staining.

### Immunohistochemistry (IHC)

Formalin-fixed paraffin-embedded xenograft tumors 4μm thick were dewaxed in xylene, hydrated in graded alcohols, and washed with PBS. After blocking endogenous peroxidase activity with 3% H_2_O_2_ aqueous solution for 10 min, the sections were incubated with primary antibodies overnight. After washing with PBS, they were then incubated with general-type IgG-HRP Polymer (Beijing CoWin Biotech Co) for 10min, followed by 3, 3′-diaminobenzidine (DAB) for about 2 to 5min. Finally, the sections were restained with hematoxylin for 1 min and then dehydrated in graded alcohols, cleared in xylene, and covered with coverslips. We adopted the H-score system developed by Hirsch et al. and used by Pirker et al. in the FLEX study [44, 45] to calculate a score of intensity multiplied by the percentage of stained tumor cells. We used rabbit polyclonal to EGFR (1:100), pEGFR [pY1068](1:300), AKT (1:2000), pAKT [pS473](1:200), ERK (1:200), pERK [Thr202/Tyr204](1:400) as primary antibodies.

### Statistical analysis

All statistical analyses were performed using SPSS version 17.0 software (SPSS, Inc., Chicago, IL, USA). Results were presented as the means ±standard deviation (SD) or Standard Error of Mean (SEM) of 3 separate assays. Differences between the different groups were assessed using a *t*-test (two-tailed). Cumulative survival was evaluated using the Kaplan-Meier method, and differences were assessed using the log-rank test. To determine independent prognostic factors, a Cox multivariate regression analysis was used. A *P* value < 0.05 was considered to indicate statistical significance.

## SUPPLEMENTARY MATERIAL TABLE AND FIGURES


