# Supplementary material for: Long non-coding RNA UCA1 induces non-T790M acquired resistance to EGFR-TKIs by activating the AKT/mTOR pathway in EGFR-mutant non-small cell lung cancer
Source: Oncotarget. 2015 Jun 8;6(27):23582–93. doi: 10.18632/oncotarget.4361 (PMC4695138; doi:10.18632/oncotarget.4361)
Supplement: Supplementary file 1 [file oncotarget-06-23582-s001.pdf]

Long non-coding RNA *UCA1* induces non-T790M acquired resistance to EGFR-TKIs by activating the AKT/mTOR pathway in *EGFR*-mutant non-small cell lung cancer

Supplementary Material

Supplementary Table S1. Progression free survival (PFS) cut-off point analysis of UCA1.

Cut-off point		PFS		
		Log-Rank	HR(95%CI)	P
10	0.0081	0.03	6.185(0.809-47.265)	0.079
15	0.0124	0.047	3.667(0.834-16.117)	0.085
20	0.0192	0.047	3.667(0.834-16.117)	0.085
25	0.0285	0.059	2.638(0.86-8.089)	0.09
30	0.0408	0.028	2.759(1.01-7.538)	0.048
35	0.045	0.048	2.359(0.927-6.009)	0.072
40	0.055	0.041	2.191(0.971-4.942)	0.059
45	0.068	0.007	3.339(1.281-8.699)	0.014
50	0.087	0.241	1.807(0.635-5.139)	0.267
55	0.0924	0.591	1.342(0.437-4.127)	0.608
60	0.1267	0.581	1.352(0.44-4.156)	0.598
65	0.1683	0.636	0.712(0.163-3.112)	0.652
70	0.2242	0.636	0.712(0.163-3.112)	0.652
75	0.2588	0.195	1.39(0.307-6.294)	0.669
80	0.3134	0.754	0.73(0.095-5.622)	0.763
85	0.4357	0.941	1.078(0.138-8.402)	0.943
90	0.6095	0.941	1.078(0.138-8.402)	0.943

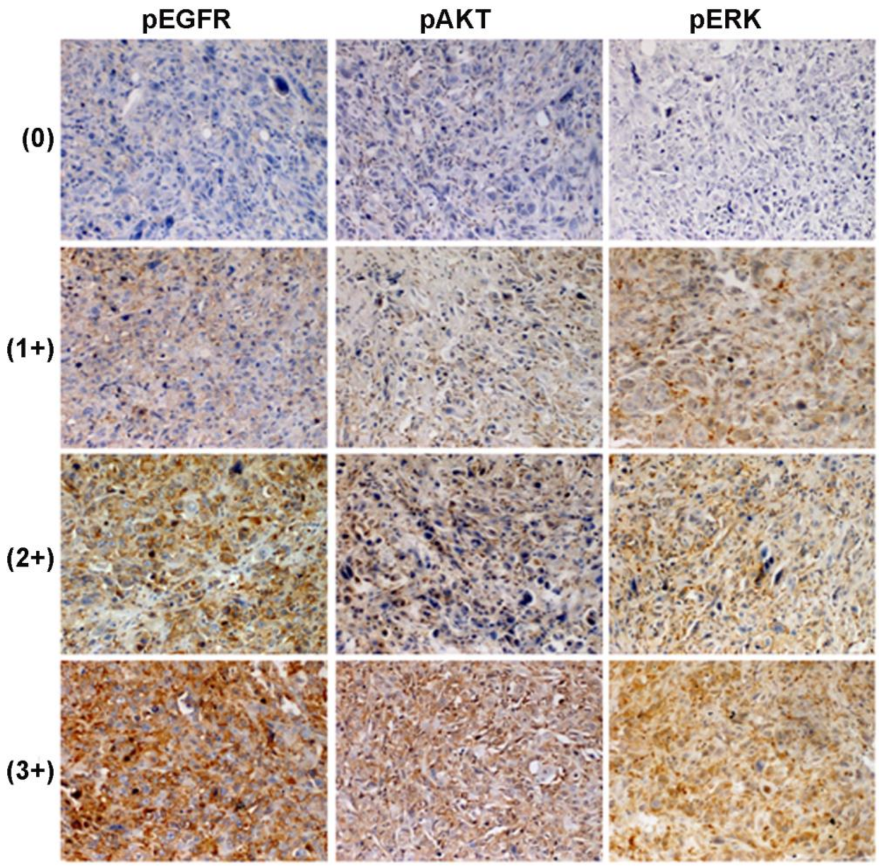

Supplementary Figure 1:

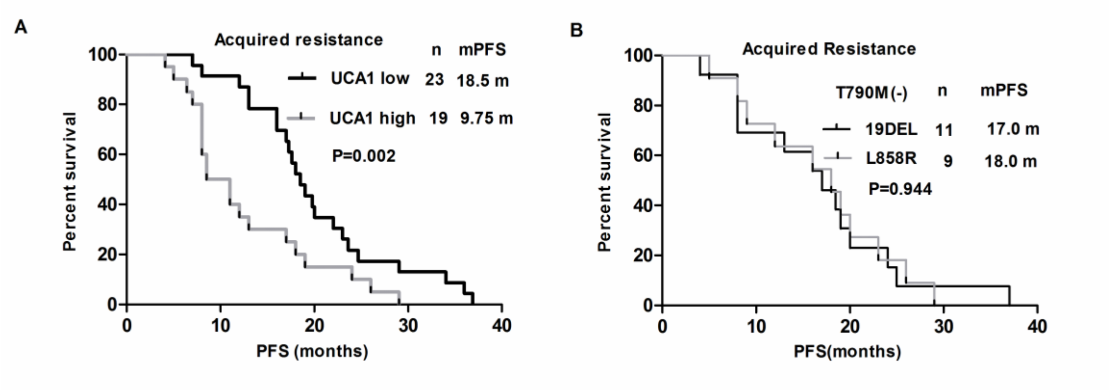

Supplementary Figure 2:

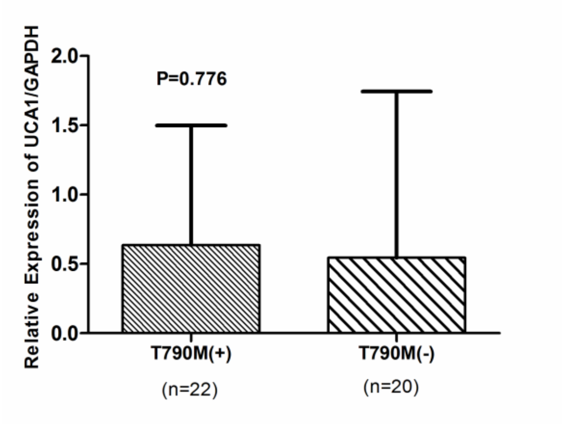

Supplementary Figure 3:

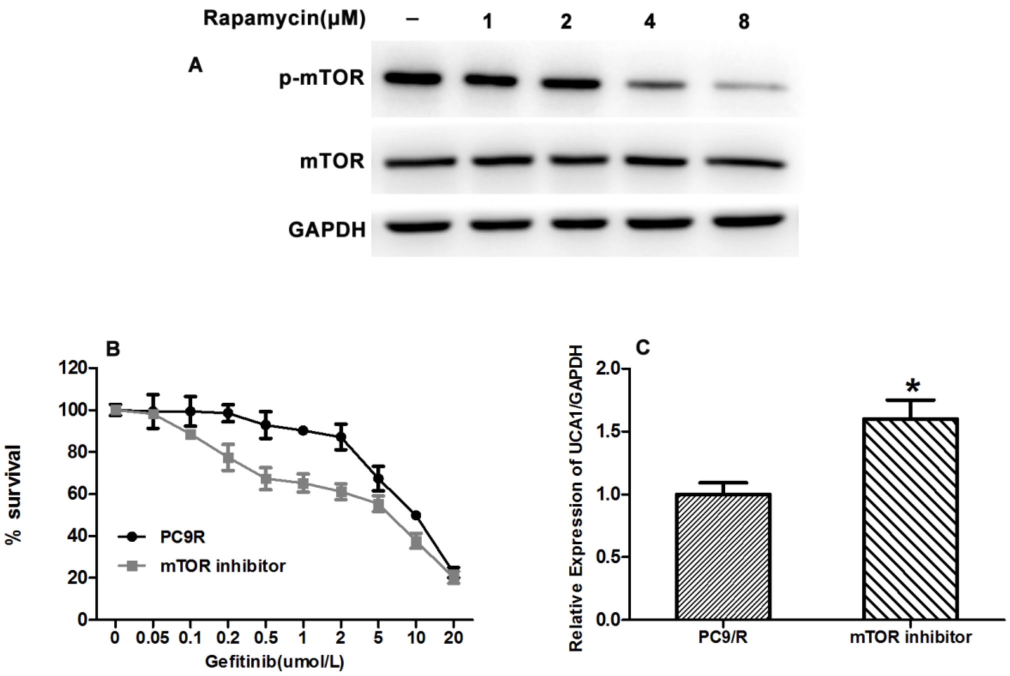

Supplementary Figure 4:
